# A community-based participatory approach to engaging Congolese migrants in intervention co-design

**DOI:** 10.1093/eurpub/ckac129.160

**Published:** 2022-10-25

**Authors:** AF Crawshaw, C Hickey, LM Lutumba, LM Kitoko, SL Nkembi, F Knights, Y Ciftci, T Vandrevala, AS Forster, S Hargreaves

**Affiliations:** 1 Institute for Infection and Immunity, St George's, University of London, London, UK; 2 Hackney Refugee and Migrant Forum, Hackney CVS, London, UK; 3 Hackney Congolese Women Support Group, London, UK; 4 Doctors of the World UK, London, UK; 5 Faculty of Health, Social Care and Education, Kingston and St George's, University of London, London, UK; 6 Our Future Health, Manchester, UK

## Abstract

**Issue:**

The World Health Organization has called for tailored, community-based interventions to address disparities in vaccination uptake affecting migrant and minoritised populations, however few exist. This study directly responds to global calls for community-centred and participatory approaches to engaging migrants in routine and COVID-19 vaccination.

**Problem description:**

Black and African migrants are known to be at risk of under-immunisation and have lower COVID-19 vaccine uptake rates in high-income countries. This UK study will use community-based participatory approaches to engage Congolese migrants in co-developing a tailored intervention to increase vaccine uptake. A community-academic coalition will lead the study. Community members will be trained as peer researchers and financially compensated. The final output will be an intervention strategy tailored to and embedded within the Congolese migrant community.

**Preliminary results:**

The coalition held 20 hours of planning meetings and peer researcher training in 2021 and co-developed a phased study involving 1) community days with poster walls and qualitative in-depth interviews with Congolese migrants, 2) interviews and workshops with local stakeholders, and 3) co-design workshops with Congolese migrants. Following outreach and pre-engagement, approximately 80 migrants attended the community days, with more than 50 interviews and 100% left positive feedback (including: felt valued, welcomed, Congolese language recognised).

**Lessons:**

Community-academic partnerships are resource-intensive but can be an effective means to build and maintain trust required to deliver a community-based research study. Academic partners should support community partners in understanding the research process to help manage expectations and provide financial compensation for their time and effort. This study offers an innovative engagement model and study design that can be adapted to other underserved populations.

**Key messages:**

• Global policy-setting organisations have called urgently for participatory research that engages migrants in the co-production of tailored initiatives to address vaccination inequalities.

• This study uses a novel, theory-driven, participatory approach to engage with and identify barriers to vaccination in Congolese migrants and co-design a tailored strategy to increase uptake.

